# Investigation of potential rhizospheric isolate for cypermethrin degradation

**DOI:** 10.1007/s13205-012-0067-3

**Published:** 2012-05-24

**Authors:** Kriti Kumari Dubey, M. H. Fulekar

**Affiliations:** Department of Life Sciences, Environmental Biotechnology Laboratory, University of Mumbai, Santacruz (E), Mumbai, 400 098 India

**Keywords:** Rhizoremediation, Soil, Cypermethrin, *Stenotrophomonas maltophilia* MHF ENV 22

## Abstract

Rhizoremediation is the use of plant–microbe interaction for the enhanced degradation of contaminants. Rhizosphere bioremediation of pyrethroid pesticides will offer an attractive and potentially inexpensive approach for remediation of contaminated soil. The present study was done with the aim of establishment of highly effective remediation method using plant with degradative rhizosphere and isolation of naturally occurring rhizosphere associated potential degrader providing the possibility of both environmental and insitu detoxification of cypermethrin contamination. The remediation efficacy of *Pennisetum pedicellatum* was investigated using green house pot culture experiments in cypermethrin amended potting soil mix (10, 25, 50, 75 and 100 mg/kg) for periodic evaluation of changes in concentration. Total proportion of cypermethrin degraders was found to be higher in rhizosphere soil compared to bulk soil. The cypermethrin degrading strain associated with rhizosphere capable of surviving at higher concentrations of cypermethrin was designated as potential degrader. On the basis of morphological characteristics, biochemical tests and 16S rDNA analysis, isolate was identified as *Stenotrophomonas maltophilia* MHF ENV 22. Bioremediation data of cypermethrin by strain MHF ENV22 examined by HPLC and mass spectroscopy, indicated 100, 50 and 58 % degradation within the time period of 72, 24 and 192 h at concentrations 25, 50 and 100 mg/kg, respectively. This is the first report of effective degradation of cypermethrin by *Stenotrophomonas* spp. isolated from rhizosphere of *Pennisetum pedicellatum*. Rhizoremediation strategy will be of immense importance in remediation of cypermethrin residues to a level permissible for technogenic and natural environment.

## Introduction

Pesticides have made a great impact to economy by preventing and reducing agricultural losses to pests and improving yield, as well as the quality of the produce in terms of cosmetic appeal. Pesticide use in developing countries is increasing to achieve higher agricultural productivity and to enable farmers to reap the benefits of related agricultural investments. Pyrethroid class of pesticides has become an important tool for crop protection due to their ability to control unwanted insects and pathogens. Cypermethrin belongs to the fourth generation of pyrethroids (Casida 1980). Cypermethrin (±)-B-Cyano**-**(3-phenoxyphenyl)methyl(±)-*cis/trans***-**3-(2,2-dichlorovinyl) 2,2dimethylcyclopropane-carboxylate is a synthetic, pyrethroid insecticide that is available in several formulations as an emulsifiable concentrate or wettable powder, widely used to control pests in cotton, vegetable crops and household purposes.

Cypermethrin is insoluble in water and has a strong tendency to adsorb to soil particles causing surface soil contamination. According to US Environmental Protection Agency (1989) under laboratory conditions, cypermethrin degrades more rapidly on sandy clay and sandy loam soils than on clay soils and more rapidly in soils low in organic material. In aerobic conditions, its soil half-life is 2–8 weeks. Cypermethrin is more persistent under anaerobic conditions. Its soil half-life is 63 days (US Department of Agriculture, Soil Conservation Service 1990). Hydrolysis and photolysis play major roles in the degradation of cypermethrin in soil. Hydrolysis of the ester linkage is the principal degradation route and leads to the formation of 3-phenoxybenzoic acid (PBA) and cyclopropanecarboxylic acid derivatives, principally, 3-(2, 2-dichlorovinyl)-2, 2-dimethyl cyclopropanecarboxylic acid (DCVA) (Kaufman et al. 1981). Cypermethrin is also subject to microbial degradation under aerobic conditions. Increased cypermethrin persistence was observed in soil with high organic matter, high clay content, reduced microbial activity and anaerobic conditions (Chapman et al. 1981). Cypermethrin soil application at 125 g of active ingredient per hectare is detectable after 15 days which becomes below detection limit after 30 days of application (Agnihotri et al.^1986^). Cypermethrin has been reported as a highly toxic agent to fish and aquatic invertebrates (Bradbury and Coats 1989) due to its high lipoaffinity and low solubility. Cypermethrin has been classified as a possible human carcinogen by US Environmental protection agency (EPA). It adversely affects the central nervous system and causes allergic skin reactions and eye irritation. Problems arising due to toxicity and carcinogenicity are causing concerns for human health, environment and ecosystem.

To address this critical issue, different physico-chemical methods have been used over the last few decades to remove pesticide pollution from the contaminated environments. The increasing costs and low efficiency of traditional physico-chemical limit the effectiveness and use of these technologies in remediation. The use of plants and native microorganisms to degrade or remove pollutants has emerged as a powerful technology for in situ remediation. The use of rhizomicrobial populations present in the rhizosphere of plants for bioremediation is referred as rhizoremediation.

The rhizoremediation of pesticide contaminated soil involves the transformation of complex/simple forms of toxic compounds into non-hazardous forms. Plant roots have a direct influence on the composition and density of the soil microbial community known as rhizosphere effect. Root morphology and secreted exudates have fundamental impacts on the rhizosphere environment stimulating the microbial activity resulting in enhanced transformation and mineralization of contaminants in the rhizosphere. There are reports of degradation/removal of pesticides (Singh et al. 2004; Yu et al. 2003; Sun et al. 2004), heavy metals (Gaur and Adholeya 2004) and other organic hydrocarbons (Biryukova et al. 2007; Chaudhry et al. 2005; Nakamura et al. 2004; Jordahl et al. 1997) by rhizosphere remediation. Their results also evidenced the enhancement of the contaminant removal in the rhizosphere zone as compared to bulk soil.

When designing a rhizoremediation treatment, the selection of suitable plant plays a crucial role. High concentrations of pollutants can prevent almost all vegetation. The rhizosphere of the plants that are able to grow under such conditions can be rich in pollutant degrading bacteria. Physical morphology of the root becomes significant factor, features such as root width, depth, surface-to-volume ratio, total biomass and surface area which vary between and within plant species effect the successful implementation of this green technology. Grass is an attractive candidate because in grass cultivars root system is highly branched and can root deeply. Root systems take up large amounts of surrounding water, thus bringing the dissolved pollutants into the rhizosphere (Erickson 1997). *Pennisetum pedicellatum* has been reported to tolerate higher concentrations of cypermethrin (Dubey and Fulekar 2011a). Rhizospheric soil could be a good source of potential degraders of cypermethrin. The number of microorganisms in the soil and their pesticide degradation abilities could affect the rate of pesticide degradation (Yu et al. 2003). Bacterial isolates capable of metabolizing pyrethroid class of compounds are of immense importance, because of their ability to provide the possibility of both environmental and in situ detoxification.

Unfortunately, very little information is available concerning the effective technology to cleanup cypermethrin residues from environment. The aim of present study was to evaluate and establish the rhizoremediation strategy for cypermethrin contamination using *P. pedicellatum* grass and isolation of potential degrader from rhizosphere soil for their possible use in degradation of cypermethrin in soil and liquid medium. Potential degrader was identified by 16S rDNA analysis, morphological and biochemical characteristics, and further selected in the present study for bench scale bioremediation studies in liquid mineral salt medium (MSM) in the laboratory.

## Materials and methods

### Soil sampling and preparation

Soil used in the present study was collected from a depth of about 0–15 cm along the banks of Surya River, Palghar (located 100 km away from Mumbai). Soil was air dried, ground, screened through 2 mm stainless steel sieve, and characterized for its physico-chemical and microbial parameters (Dubey and Fulekar 2011a).

### Spiking of soil mix for pot experiments

Technical grade cypermethrin (94 % purity) was obtained from AIMCO Pesticides, Maharashtra, India. Experimental potting soil mix (soil, sand and VAM mixture) was spiked with cypermethrin. In the spiking procedure, 25 ml of acetone-containing pesticide was added to 25 % of the soil sample (250 g), flasks were closed for 5 min (min) to let the solvent disperse. Thereafter, the solvent was completely evaporated for 16 h at room temperature and sub-sample was mixed with the remaining 75 % (750 g) of the soil sample (Brinch et al. 2002). All samples were thoroughly mixed with a metal spatula to obtain final cypermethrin concentrations of 10, 25, 50, 75 and 100 mg/kg of dry weight of soil, unspiked soil (receiving only acetone without cypermethrin) was taken as control.

### Experimental design

#### Rhizosphere bioremediation green house experiment

The present research work has been carried out to evaluate the potential of *P. pedicellatum* for rhizosphere bioremediation of cypermethrin using pot culture experiment. Pot culture experiments were conducted in the green house where *P. pedicellatum* was grown in soil amended with cypermethrin at various doses concentrations (100, 75, 50, 25, and 10 mg/kg). The rhizosphere bioremediation of cypermethrin was carried up to the period of 60 days at the intervals of 7, 15, 30, 45 and 60 days. Pots were filled with soil mix [soil and sand mixture (<2 mm) 3: l (w/w) along with about 20 % mycorrhizal (VAM) inoculum] spiked at various concentrations. The seeds of the *P. pedicellatum*(procured from Indian grassland and fodder research institute, Zhansi) were surface sterilized with 70 % ethanol for 30 s followed by sterilization with 0.1 % mercuric chloride for 5 min. Seeds were thoroughly washed 5 times with sterile distilled water. Twenty pre-germinated seeds were transferred to plastic pots (1 kg, 11-cm diameter, 11.5-cm height) containing contaminated and control soil. Three replicates were done for this experiment and pots were kept in green house having temperature 24–26 °C at day, 22–23 °C at night with the natural light. There were six treatments in this trial, contaminated soil spiked with five concentrations of cypermethrin and unspiked soil as control. Soil samples were collected (Wollum 1982) at five intervals of 7, 15, 30 and 45 and 60 days to evaluate rhizospheric degradation of cypermethrin. The dissipation of cypermethrin in *P. pedicellatum* rhizosphere soils at different concentrations was quantified using HPLC and mass spectroscopy. The rhizospheric and bulk soil samples were also microbial enumerated (immediately within 24 h of the final harvests) at the end of experiment. Plate counts of bacteria were taken by spread plate method and compared. Total microbial numbers were evaluated on nutrient agar medium while degrading microbial numbers were evaluated on MSM agar medium supplemented with cypermethrin (50 μg/ml).

#### Data analysis for degradation kinetics of cypermethrin in soil

Log residues of cypermethrin were plotted against respective days to determine the kinetics model of degradation. It was found to produce straight line for all treatment doses, thus following the first-order kinetics model. Therefore, degradation rate constant (*k*) using Eq. (1) and half-life using Eq. (2) were determined using first-order kinetics model.1where *C*_0_ is the initial concentration of cypermethrin (mg/kg) at time zero, *C*_*t*_ is the concentration of cypermethrin (mg/kg) at time *t*, and *k* and *t* are the rate constant (day^−1^) and degradation period in days, respectively. The half-life (T1/2) of cypermethrin at different doses was calculated using the algorithm as expressed in (Eq. 2).2where *k* is the rate constant (day^−1^).

#### Extraction and clean up of soil samples

For analysis, soil from pots was carefully collected, mixed and each replicated soil sample (10 g) was added with activated charcoal (0.05 g), florisil (0.05 g) and anhydrous sodium sulfate (1 g) and mixed well. The mixture was packed in filter paper to fit in a soxhlet apparatus for extraction with 150 ml of hexane: acetone solvent mixture (1:1 v/v) for 6 h. The extracted solvent was then passed through anhydrous sodium sulfate and concentrated. The residue was then dissolved in methanol (10 ml) for final estimation of cypermethrin.

#### Isolation of potential degrader from rhizoremediated rhizospheric soil

The rhizoremediated soil initially amended with higher concentration of cypermethrin (100 mg/kg) obtained from the first set of experiment was used for the isolation of potential degrader. Rhizosphere soil samples from triplicate pots and a composite sample was prepared for the highest concentration i.e.100 mg/kg. Microorganism that could utilize cypermethrin as a sole carbon source was isolated using mineral salt medium (MSM) in the enrichment study (Siddique et al. 2003). MSM was used in both enrichment culture of soils and liquid culture of isolated bacteria. The composition of MSM used in this study was prepared to assess pesticide cypermethrin as a sole carbon source for microorganism in the enrichment study. The composition of MSM (mg/L): K_2_HPO_4_ 255, KH_2_PO_4_ 255, (NH_4_)_2_SO_4_ 255, MgSO_4_·7H_2_O 50, CaCO_3_ 5, and FeCl_2_·4H_2_O 5, blended with 1 ml of trace elements solution. The trace element solution contained (in mg/L): MgSO_4_·H_2_O 169, ZnSO_4_·7H_2_O, 288, CuSO_4_·5H_2_O 150, NiSO_4_·6H_2_O 260, CoSO_4_, 280 and Na_2_·MoO_4_·2H_2_O 240. A 10 g soil sample was homogenized in 10 ml of 0.85 % saline solution by shaking the preparation on a rotary shaker at 28 °C and 150 rpm. A tenfold dilution series was prepared and 0.1 ml of each dilution was inoculated into test tubes containing 3 ml of MSM supplemented with 50 μg/ml of cypermethrin as the sole carbon source. The tubes were incubated on a rotary shaker at 28 °C for 4 weeks at 150 rpm. The culture of the terminal positive tube showing the growth was enriched by two serial transfers into fresh media. MSM agar medium was used for the purification and isolation of degrading bacterial strains. Each enriched culture was streaked onto MSM agar plates. Five bacterial colonies were obtained on MSM agar. Each colony was further subjected to increasing concentration of cypermethrin in MSM (50, 75 and 100 mg/L). Bacterial colony surviving at highest concentration was designated as potential degrader.

#### Taxonomic identification of the potential cypermethrin degrading strain

Visually distinct colonies of potential degrader were picked up, purified and selected for identification from the minimal agar media plates (supplemented with cypermethrin as the sole source of carbon). Identification was performed using Gram staining kit (K001-1KT, Hi-Media) and observed under the light microscope, standard physiological and biochemical tests were performed using KB003 kit (KB003 Hi25, Himedia, India). Further, the genomic DNA was extracted (Sambrook and Russel 2001) and 16S rDNA was PCR amplified (Weisburg et al. 1991) using universal primers 8F: 5′ AGA GTT TGA TCC TGG CTC AG 3′, 1492R: 5′ ACG GCT ACC TTG TTA CGA CTT 3′ according to Dubey and Fulekar (2011b). The PCR product was sequenced and the data were aligned and analyzed on BLAST database to identify the bacteria and their closest neighbors. The 16S rDNA sequence used for phylogenetic analysis was compared with the other 16S rDNA bacterial sequences available in EMBL/Genbank database. Phylogenetic tree was constructed using neighbor joining method. Novel potential degrader was identified as *Stenotrophomonas maltophilia* MHF ENV 22 (GenBank accession number HQ661377.1) which further merits the selection for bioremediation studies.

#### Bioremediation of cypermethrin by potential degrader

##### Bioremediation of cypermethrin

*Stenotrophomonas maltophilia* MHF ENV 22 was taken for bioremediation studies in liquid MSM. Purity of bacterial colonies was checked at regular intervals on MSM agar (2 %) supplemented with cypermethrin. The inocula for all of the bioremediation experiments were prepared by growing bacteria in 50 ml of MSM at 28 °C on a rotary shaker at 150 rpm containing cypermethrin (50 μg/ml) for 48 h. Cell pellets were harvested by centrifugation at 6,000*g* for 10 min. Cells were washed three times with 25 ml of sterile 0.0125 M phosphate buffer (pH 7.2) and quantified by the dilution plate count technique. For all experiments, 10^6^ cells/ml were used and samples were incubated at 28 °C at 150 rpm. Cell concentration was determined by measuring the optical density at 600 nm (OD 600) and converting it to colony forming unit (CFU)/ml using a standard calibration curve. For spiking of cypermethrin to Erlenmeyer flasks (250 ml), 1 ml acetone containing required cypermethrin concentration was aseptically added to autoclaved Erlenmeyer flasks allowing the acetone to evaporate. After complete evaporation of acetone from the Erlenmeyer flasks, 100 ml sterile culture media were added under laminar flow hood so as to reach the desired final concentration of organic compound. Biodegradation was assessed by comparing the disappearance of cypermethrin in samples and controls over time. Sterile controls containing cypermethrin were prepared for each concentration to discern solubilization and adsorption losses. Bioremediation of cypermethrin was carried out by MHF ENV 22 in 250 ml Erlenmeyer flasks containing 100 ml MSM at 28 °C and continuous shaking at 150 rpm. In batch biodegradation experiment, 25, 50, and 100 mg/L cypermethrin were taken as a single carbon source in 100 ml of mineral media. Samples were withdrawn periodically to assess biodegradation by comparing the cypermethrin concentrations in sample and controls over time. Samples were withdrawn similarly transferred to 10-ml vials and capped with teflon-coated septa for HPLC analysis. Optical density (600 nm) of the samples was also determined for monitoring the growth and proliferation of the microorganism during the bioremediation experiment. Variation in biological oxygen demand (BOD) and chemical oxygen demand (COD) was also observed as indicators of bioremediation.

#### Extraction of cypermethrin from MSM for analysis

For degradation studies of cypermethrin in liquid medium, samples were centrifuged (10 min, 10,000 rpm, Plasto crafts, Rota 6R—V/Fm) to separate cell mass and the supernatant and extracted with equal volume of *n*-hexane by vigorous shaking for 15–20 min in a separatory funnel. The hexane layer was separated and evaporated to dryness at 70 °C using vacuum rotary evaporator. The dried residue was then dissolved in 10 mL HPLC grade methanol and was used for analysis. Each sample was injected 3 times and the mean was calculated. The results of cypermethrin concentration remained in soils at five harvesting periods (i.e. 7, 15, 30, 45 and 60 days) for all treatments were calculated and expressed in mg/kg dry weight of soil.

#### Instrumentation

The samples were injected in high pressure liquid chromatography (HPLC, Shimadzu, Japan) system consisted of a solvent delivery pump LC-10 AS, connected with an autoinjector model SIL-6A, rheodyne injection valve fitted with a sample loop (20 ml) and C-18 column. Effluents were monitored using UV-detector (visible spectrophotometer detector SPD-10A). The output of the detector was connected to a chromatopack (CR6A). Mobile phase consisted of methanol (Merck HPLC grade) since cypermethrin is miscible in alcohol. The methanol was purified by filtration through Millipore filtration unit (0.2 and 0.4 mm Millipore filter, micropore, nylon). The filtered methanol was degassed prior to use by sonication. The flow rate was adjusted at 2 mL/min with total elution time of 10 min for each run. The column was flushed with deionized distilled water and methanol for removing impurities and was allowed to equilibrate between runs. Cypermethrin was further identified by comparing UV spectra and retention times with the standards. The samples were also analyzed by mass spectrometry to identify cypermethrin and its metabolites using *m*/*z* spectra.

## Results and discussion

### Rhizoremediation of cypermethrin using green house pot culture experiment

**Fig. 1:**
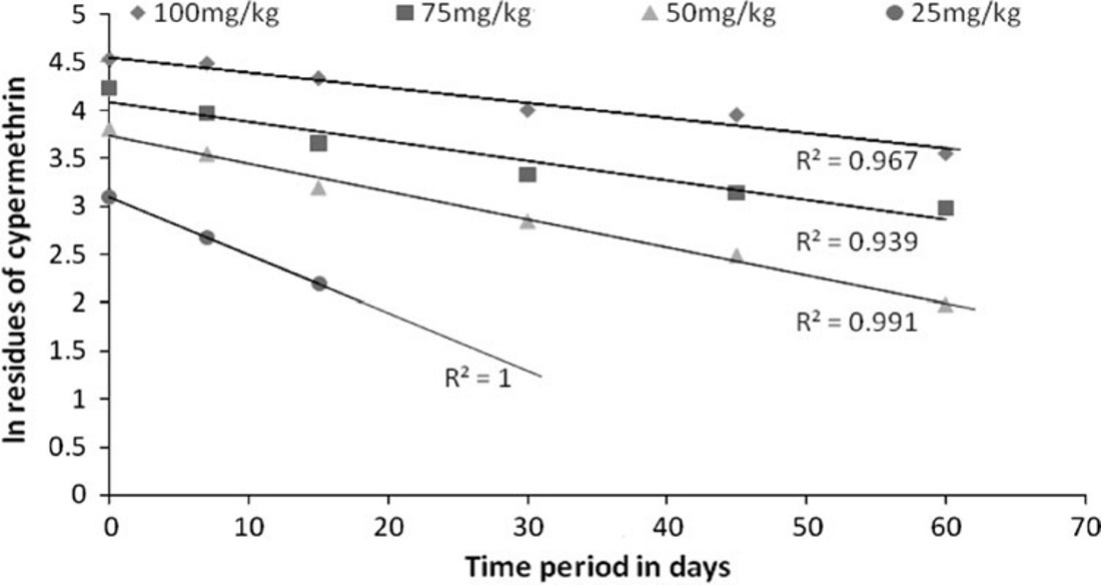
First order reaction kinetics of rhizospheric dissipation of cypermethrin

**Fig. 2:**
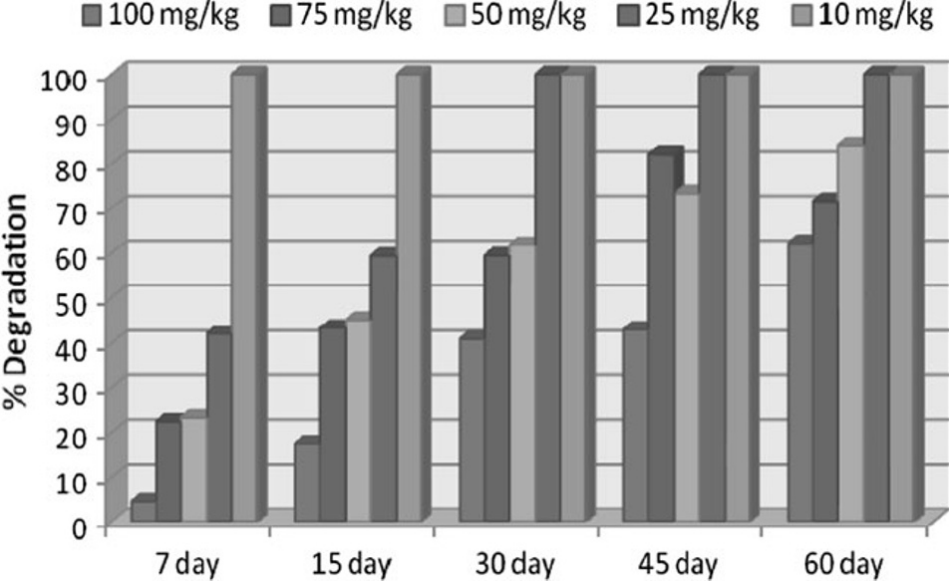
Percentage degradation of cypermethrin during rhizoremediation by *P. pedicellatum*

Rhizospheric degradation results indicated lower degradation percentage at higher doses 100, 75 and 50 mg/kg in comparison to 50 and 25 mg/kg in the order of 25 > 50 > 50 > 75 > 100 mg/kg. The dissipation followed first-order reaction as the ln values of the residues of cypermethrin (*C*_t_/*C*_0_) when plotted against respective days produced straight line for all the treatment doses (Fig. 1) following the first-order degradation kinetics. The residue data were, therefore, statistically interpreted for computation of regression equation and residual half-life values (Table 1). The mean extraction recovery of cypermethrin achieved from spiked soil is reported in Table 1. The initial recovered concentrations of cypermethrin were 92.6, 92.0, 90.0, 88.4, 80.0 mg/kg for 100, 75, 50, 50, and 25 mg/kg doses, respectively, which dissipated to 4.42 and 62.21 % at the harvest period of 15 and 45 days, respectively, in case of 100 mg/kg, and NDL(non-detectable limit) on 60th day after application. The initial deposit in 75 mg/kg was dissipated by 41.6 % on 15th day that progressively increased to 82.5 % on 45th day and NDL after 60 days. The corresponding values were about 27.7, 47.95 and 75.52 % and NDL, respectively, on 7th, 15th and 30th days in 50 mg/kg. The percentage dissipation was 34.7, 53.03 % and NDL in 50 mg/kg on 7th, 15th and 30th days, respectively, 25 mg/kg was found to be completely degraded at the first harvest period of 7 days (Fig. 2). The rate of dissipation as indicated by the regression coefficient further substantiated the findings that the persistence of cypermethrin increased with increased initial concentration in soil. The half-life values in the present experiment were found to vary from 11.55 to 43.31 days for the treatment doses.

**Table 1:** Degradation kinetics data of cypermethrin in soil

Dose (mg/kg)	Mean extraction	7 days	15 days	30 days	45 days	60 days	T1/2 days	Regression equation
100	92.60	88.50	76.40	54.70	52.80	35.0	43.31	4.557 − 0.016*X*
75	68.90	53.50	39.0	28.0	23.31	19.80	34.65	4.085 − 0.020*X*
50	45.0	34.50	24.32	17.23	12.0	7.2	23.89	3.734 − 0.029*X*
25	22.10	14.48	9.0	NDL	NDL	NDL	11.55	3.09 − 0.06*X*
10	8.10	NDL	NDL	NDL	NDL	NDL	–	–

*NDL* non detection limit

There are few reports on the degradation of pyrethroid insecticides in soils (Kaufman et al. 1981; Roberts and Standen 1981). The synthetic pyrethroid insecticides cypermethrin is immobile in soil and cannot be readily leached through soil. The leaching potential of synthetic pyrethroids in soil has been reported to be very limited due to their significant higher hydrophobicity and resulting strong adsorption to soil (Kaufman et al. 1981). Dissipation of cypermethrin can be attributed to *P. pedicellatum* rhizosphere and associated microbial microflora. Microbial degradation is known to be one of the most important factors determining the environmental fate of pesticide in the soil (Arnold 1990). Wauchope et al. (1992) reported the half-life of cypermethrin to vary from 4 days to 8 weeks. Chai et al. (2009) studied the degradation of cypermethrin in topsoil and found that degradation followed first-order degradation kinetics. Degradation of cypermethrin in soil significantly depends on either soil characteristics or microbial activity (Miyamoto 1981). 3-Phenoxy benzoic acid was majorly detected in the rhizosphere soils initially spiked at 75 and 100 mg/kg (higher) concentrations suggesting that 3-phenoxy benzoic acid formed in the soils contaminated at lower levels dissipated quickly into simpler compounds which thus became below detectable limits. 3-Phenoxy benzyl alcohol was also detected during rhizospheric dissipation, which is of low mobility, direct hydrolysis product of permethrin degradation, and a metabolite of 3-phenoxy benzoic acid produced during degradation of cypermethrin (Kaufman et al. 1981). Formation of 3-phenoxy benzyl alcohol and 3-phenoxy benzoic acid confirms the major degradation pathway of cypermethrin via the hydrolytic cleavage of an ester linkage (Miyamoto 1981). 3-phenoxy benzoic acid is a degradation product of cypermethrin, produced during microbial metabolism of 3-phenoxy benzyl alcohol in soil (Kaufman et al. 1981). Grant et al. (2002) also reported that soil-bacteria are able to degrade this insecticide belonging to genera *Pseudomonas* and *Serratia*. Therefore, *P. pedicellatum* itself, in addition to the rhizosphere bacterial consortium, seemed to play an important role in reducing the cypermethrin level in the soil. Due to high cypermethrin tolerance (Dubey and Fulekar 2011a) and rhizospheric dissipation capability of *P. pedicellatum* makes this plant suitable for decontamination and remediation of contaminated sites.

### Isolation and identification of potential degrader from rhizosphere

The experiment carried out in the first set of experiment proved the effectiveness of the *P. pedicellatum* plant in bioremediation of cypermethrin from soil. For isolation of potential degrader, rhizospheric and bulk soil was collected, bacterial enumeration was done at the end of pot culture green house experiment and compared. The magnitude of bacterial population was found to be higher in rhizospheric soil samples as compared to bulk soil for all the treatment dosage 100, 75, 50, 50 and 25 mg/kg (Table 2). This may be due to selective enrichment of cypermethrin degraders in the rhizospheric zone. The soil in the rhizospheric zone generally consists of 10–100 times greater number of indigenous microorganisms than in bulk soil, this might be due to carbon containing compounds exudated from plant root such as sugars, carbohydrate, alcohol and amino acids, which could stimulate the microbial activities and increase the number of rhizosphere microorganisms (Schnoor et al. 1995; Stottmeister et al. 2003). The pesticide degradation ability was found to be increased in this soil (Anderson et al. 1994). The microbial consortium present in rhizoremediated soil was found to be bacterial genera (*Alcaligenes* sp., *Bacillus* sp., *E. coli* sp., *Pseudomonas* sp., *Rhodococcus* sp., *Serratia* sp., *Stenotrophomonas* sp., *Streptococcus* sp.) and fungal genera (*Aspergillus flavus, Aspergillus fumigatus, Penicillium* sp., *Rhizopus* sp, *Mucor* sp.). Rhizospheric soil samples of highest concentration 100 mg/kg of rhizoremediated soil were selected for isolation of potential degrader at the end of green house experiment. After 4 weeks of enrichment, bacterial strains in rhizosphere were isolated and purified. The potential microorganism existing in rhizospheric soil which has capacity to survive and multiply at higher concentration of cypermethrin was further selected, identified and characterized for bioremediation of this hazardous compound.

**Table 2:** Microbial numbers in rhizoremediated bulk and rhizospheric soils (mean values ± SD) at different doses of cypermethrin

Concentration of cypermethrin in mg/kg	Log CFU^#^/g soil			
	Total microbial numbers		Cypermethrin degrading microbial numbers	
	Bulk	Rhizosphere	Bulk	Rhizosphere
Control	5.43 ± 1.08	6.95 ± 1.37	ND*	ND
10	5.75 ± 1.38	6.83 ± 2.25	1.20 ± 1.21	1.82 ± 1.10
25	5.11 ± 1.73	6.23 ± 2.52	1.61 ± 0.98	2.16 ± 1.89
50	4.89 ± 1.73	6.02 ± 2.64	2.62 ± 0.70	3.02 ± 0.29
75	4.45 ± 2.24	5.98 ± 1.22	3.72 ± 1.92	4.21 ± 0.98
100	4.26 ± 2.11	5.23 ± 0.74	4.53 ± 1.72	5.98 ± 1.34

*CFU*^*#*^ colony forming unit

ND* not detected

In order to identify the isolated potential degrader, different morphological, physiological and biochemical tests were conducted. Isolate was gram-negative rod with positive reaction (more than 90 %) for ornithine decarboxylation, urease activity, citrate utilization, malonate utilization and catalase production, partially positive reaction (11–89 % positive) for trehalose and glucose utilization and negative reaction (more than 90 %) for phenylalanine deaminase, nitrate reduction, H_2_S production, Voges-proskauer’s test, Methyl red test, Indole test, Esculin hydrolysis, and utilization tests for arabinose, rhamnose, cellobiose, melibiose, saccharose, raffinose, lactose. These biochemical characteristics indicated that isolate belonged to the genus *Stenotrophomonas*.

**Fig. 3:**
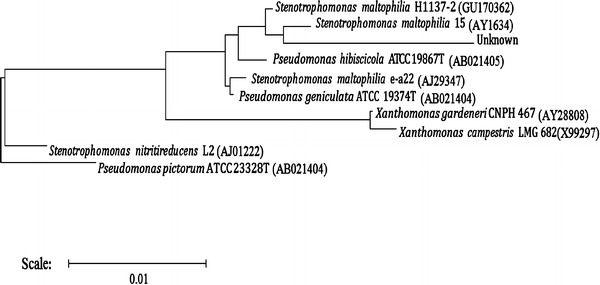
Phylogenetic tree of potential degrader isolated from rhizoremediated soil (based on 16S rDNA sequence analysis). Unknown refers to *S. maltophilia* MHF ENV 22, the current strain reported in this study

For further phylogenetic characterization, the genomic DNA of bacteria was isolated, and 16S rDNA was amplified using universal primers. The PCR product was sequenced and aligned using Clustal W software. The 16S rDNA sequence obtained was used for phylogenetic analysis and compared with the other 16S rDNA bacterial sequences available in EMBL/Genbank database. The BLAST search of available data in the EMBL/Genbank database showed a high similarity (99 %) with *S. maltophilia* (Fig. 3). The 16S rDNA sequence of *S. maltophilia* MHF ENV22 was deposited in GenBank database under accession number HQ661377.1. Though member of genus *Stenotrophomonas* has been isolated and studied from many environments, their close association with plants has been noted. *Stenotrophomonas* spp. also have promising applications in bioremediation and phytoremediation, as these bacteria can metabolize a large range of organic compounds that are present in the rhizosphere. Chen et al. (2011a, b) also reported *Stenotrophomonas* sp. strain ZS-S-01 for bioremediation of cypermethrin.

Root morphology and secreted exudates have fundamental impacts on the rhizosphere environment and its microbial community. In the rhizosphere, plant root exude compounds that can serve as co-metabolites in microbial pollutant degradation (Hedge and Fletcher 1996). Apart from the direct release of degradative enzymes, the susceptibility of organic pollutants to bacterial metabolism and enzymatic attack differs widely and is related to molecular structure. Plants are able to stimulate the activities of microbial degrader organism/communities. Mechanism of nonspecific stimulation potentially involved in rhizodegradation includes exudates that serve as analogs or co-metabolites of organic pollutants (Siciliano and Germida 1998). Cypermethrin amendment to soil offers limits of tolerance of the plant and microbial association and enables selective enrichment of cypermethrin degrading potential bacterial strain which was further isolated in pure form and tested for the cypermethrin utilizing capacity in the laboratory.

### Bioremediation of cypermethrin by potential degrader *S. maltophilia* MHF ENV 22

**Fig. 4:**
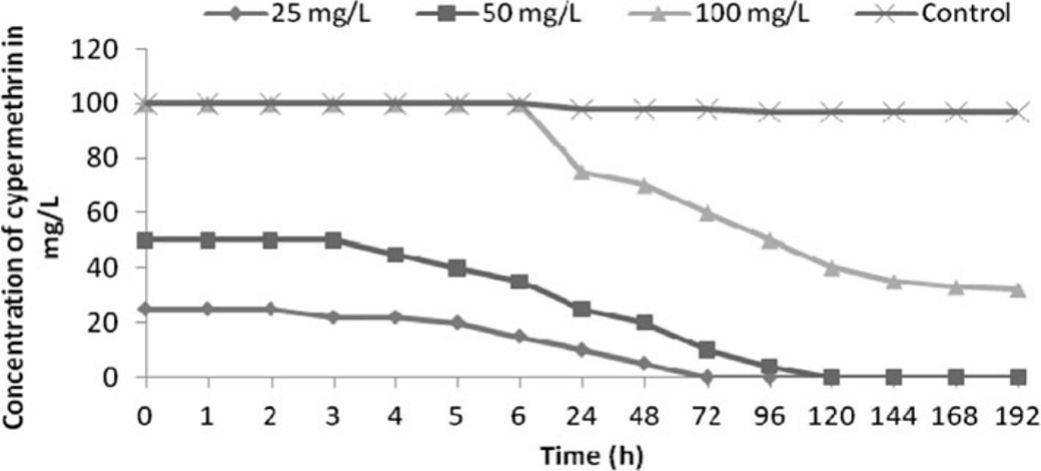
Concentration of cypermethrin during bioremediation by *S. maltophilia* MHF ENV 22

**Fig. 5:**
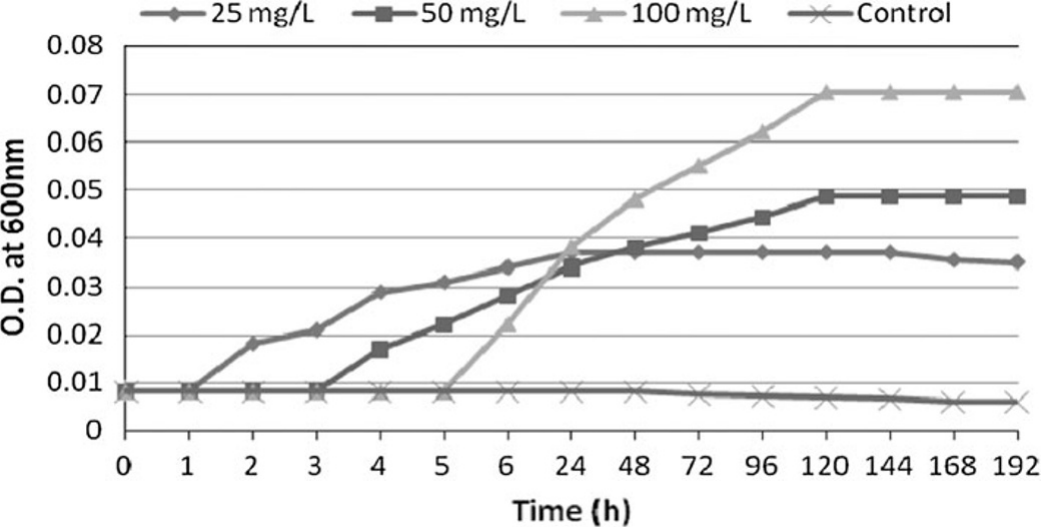
Measurement of optical density (OD) at 600 nm during cypermethrin bioremediation by *S. maltophilia* MHF ENV 22

*Stenotrophomonas maltophilia* MHF ENV 22 colonies on MSM agar plates were circular, smooth, glossy, convex, pale yellow and 0.8–1.2 mm in diameter after 3 days incubation at 28 °C. In microscopic examination, its cells were straight, rod-shaped, gram-negative, motile, 0.3–0.5 μm in diameter and 1.3–1.8 μm in length. In the bioremediation experiment, initial concentrations 25, 50 mg/l, 100 mg cypermethrin were taken. The experimental findings indicated isolated *S. maltophilia* strain started biodegradation of 25 mg/L cypermethrin within 2 h and degraded it completely within 48 h. For 50 mg/L cypermethrin, a lag phase of 5 h was observed, degradation was started from 6 h and 50 % was degraded within 96 h. Similarly, 100 mg/L cypermethrin was consumed 66.6 % after 96 h by *S. maltophilia* MHF ENV 22 (Fig. 4). A lag phase of 6 h was observed in this case (Fig. 5). Earlier a bacterial strain *Ochrobactrum lupini* DG-S-01 was found to be capable of maximum cypermethrin degradation over 90 % of the initial dose of beta-cypermethrin (50 mg/ L) within 5 days at 30 °C and pH 7.0 in MSM supplemented with glucose, beef extract and yeast extract (Chen et al. 2011a, b). *Pseudomonas aeruginosa* CH7 (capable of biosurfactant production) has been reported for 90 % biodegradation of beta-cypermethrin within 12 days with inocula biomass of 0.1–0.2 g/ L at 25–35 °C, pH 6–9, and a final concentration of beta-cypermethrin 25–900 mg/ L (Zhang et al. 2011). Two *Serratia* strains JC1 and JCN 13 under their own optimal degradation conditions were found to degrade 92 % beta-cypermethrin within 10 days and 89 % within 4 days, respectively. High Cell surface hydrophobicity of strain JC1 was credited for enhanced degradation of cypermethrin (Zhang et al. 2010).

OD was found to be increasing along the concentration of cypermethrin within a period of 192 h experiment and remained constant as the incubation progressed towards the end of the experiment. These research findings indicate increased degradation of cypermethrin by isolated potential degrader. The increase in OD is characteristically from bacteria growing under shaking using cypermethrin as the sole carbon source in amounts exceeding the aqueous solubility in the MSM at selected concentrations of 25, 50 and 100 m/l. Microorganisms need an acclimatization period to induce the formation of necessary degradative enzymes. This may account for prolonged lag phase which was observed at higher concentration of cypermethrin. Our findings are in accordance with Jilani and Khan (2006) who also reported that increased concentration of cypermethrin has a marked effect on biodegradation performance of IES-*Ps*-1 with a modest increased in the duration of lag phase of IES-*PS*-1, capable of 82 % cypermethrin degradation at 40 mg/L dose in approximately 48 h. As the concentration was increased to 80 mg/L, 50 % degradation of this compound was observed which reduced to 17 % at 125 mg/L, respectively.

**Fig. 6:**
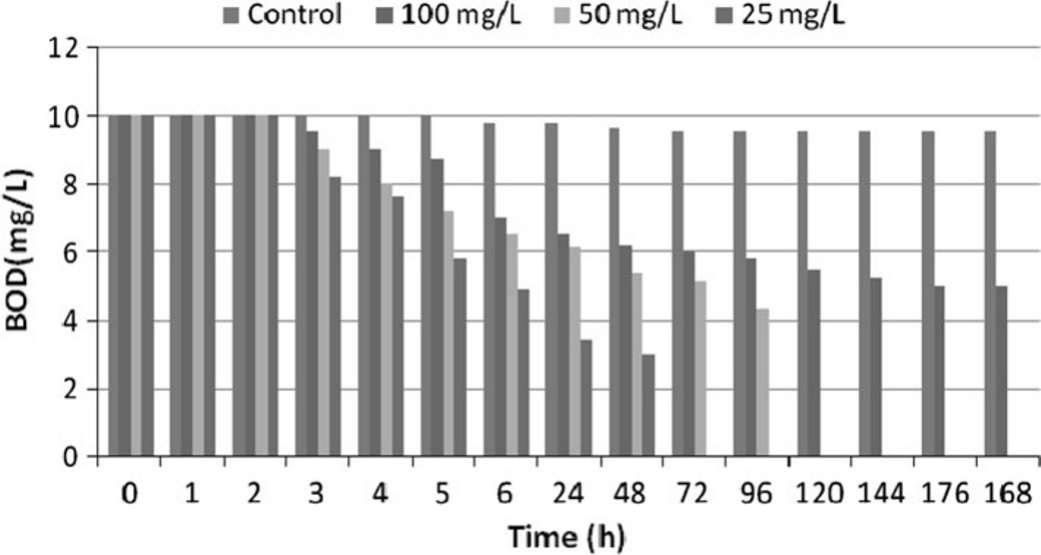
Biological oxygen demand (BOD) variations during cypermethrin bioremediation by *S. maltophilia* MHF ENV 22

**Fig. 7:**
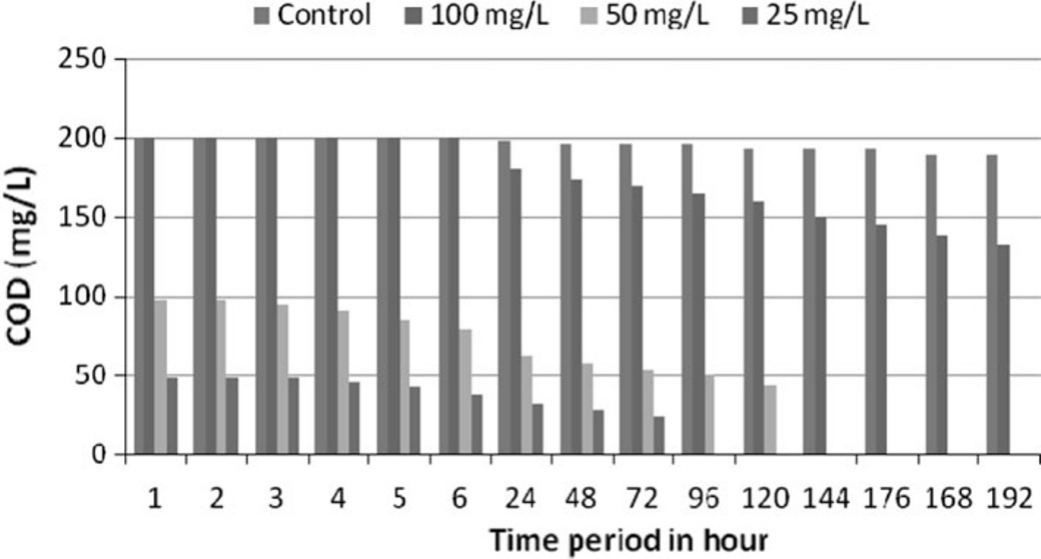
Chemical oxygen demand (COD) variations during cypermethrin bioremediation by *S. maltophilia* MHF ENV 22

In the initial phase, exponential growth was based on cypermethrin dissolved in the aqueous medium, close to or at the maximum concentration. In this phase, population growth was controlled only by metabolic activity, and not by cypermethrin availability. Exponential growth ceased when cypermethrin consumption by the isolates exceeded the cypermethrin dissolution rate. Isolates reached a pseudo-linear growth phase when limited physically by the maximum dissolution of cypermethrin which is converted in cells. In the pseudo-stationary phase, the cypermethrin consumption of individual cells reached the maintenance level and consequently the growth ceased. The increase in cell count of isolates during degradation was an indication that the cypermethrin supported microbial biomass product as a sole source of carbon and energy. Researchers have shown that population density increase in media was a reflection of degradation process and proliferation of cell mass (Kuruode et al. 2005). As such the increase in population density of the isolates in the experimental media compared to the control as degradation proceeded was an indication of metabolic activity reflected in the increased cell count. During bioremediation biological oxygen demand (BOD) and chemical oxygen demand (COD) were also monitored as indicators for microbial growth and bioremediation. The decrease in BOD values indicates the growth of microorganisms in the varying concentration of cypermethrin (Fig. 6). The basis for COD test is that nearly all organic compounds can be fully oxidized to carbon dioxide with a strong oxidizing agent under acidic conditions. Figure 7 demonstrates the decrease in COD levels over a period of bioremediation, which indicates degradation of cypermethrin by strain MHF ENV 22. During bioremediation decrease in biological oxygen demand (BOD) and chemical oxygen demand (COD) have been reported as indicators for microbial growth and bioremediation (Singh and Fulekar 2010). Bioremediation samples were analyzed on mass spectrometer for detection of its metabolites or intermediates. No persistent accumulated metabolite was observed during bioremediation studies of cypermethrin at the concentrations 25, 50, and 100 mg/l. The mass spectrometry analysis of bioremediation samples showed the presence of phenol (*m*/*z* 95) and catechol (*m*/*z* 109) during bioremediation (Table 3) confirming the biodegradation of cypermethrin by *Micrococcus* sp. strain CPN 1 via ortho cleavage pathway (Tallur et al. 2008). Degradations reactions involved conversion of cypermethrin to 3-phenoxy benzoate which was found to be further metabolized to protocatechuate and phenol which is in accordance with the degradation pattern of *Pseudomonas pseudoalcaligenes* POB310 (Halden et al. 1999) and *Micrococcus* sp. strain CPN 1(Tallur et al. 2008). The pesticide degrading potential of a bacterial culture isolated from rhizosphere was examined with the aim of isolation and characterization of cypermethrin degrading strain possessing degradability in liquid media as well.

**Table 3:** Chromatographic properties of cypermethrin metabolites during bioremediation

Code	*m*/*z*	Compounds
A	109	Catechol
B	95	Phenol
C	141	2-Hydroxy-muconic- semialdehyde

## Conclusion

Our findings indicated that Rhizosphere bioremediation will offer a particularly attractive, potentially inexpensive and effective approach to remove cypermethrin residues from soil using *P. pedicellatum*. In addition, amendment of cypermethrin to soil improve selective enrichment of cypermethrin degrading indigenous rhizosphere microorganisms, thus rhizospheric soil can act as a good source of potential degrader. The isolated potential degrader *S. maltophilia MHF* ENV 22 will offer an inexpensive approach of bioremediation for cypermethrin contaminated sites.
